# A Holistic Perspective on How Photobiomodulation May Influence Fatigue, Pain, and Depression in Inflammatory Bowel Disease: Beyond Molecular Mechanisms

**DOI:** 10.3390/biomedicines11051497

**Published:** 2023-05-22

**Authors:** E-Liisa Laakso, Tatjana Ewais

**Affiliations:** 1 Mater Research Institute-University of Queensland, South Brisbane, QLD 4101, Australia; 2 Menzies Health Institute Queensland, Gold Coast Campus, Griffith University, Southport, QLD 4215, Australia; 3 Mater Adolescent and Young Adult Health Clinic, South Brisbane, QLD 4101, Australia; tatjana.ewais@mater.org.au; 4 School of Medicine, The University of Queensland, St Lucia, QLD 4068, Australia; 5 School of Medicine and Dentistry, Gold Coast Campus, Griffith University, Southport, QLD 4215, Australia

**Keywords:** Crohn’s disease, ulcerative colitis, integrated mechanism

## Abstract

Background: Numerous mechanisms, mostly molecular, have been tested and proposed for photobiomodulation. Photobiomodulation is finding a niche in the treatment of conditions that have no gold-standard treatment or only partially effective pharmacological treatment. Many chronic conditions are characterised by symptoms for which there is no cure or control and for which pharmaceuticals may add to the disease burden through side effects. To add quality to life, alternate methods of symptom management need to be identified. Objective: To demonstrate how photobiomodulation, through its numerous mechanisms, may offer an adjunctive therapy in inflammatory bowel disease. Rather than considering only molecular mechanisms, we take an overarching biopsychosocial approach to propose how existing evidence gleaned from other studies may underpin a treatment strategy of potential benefit to people with Crohn’s disease and ulcerative colitis. Main findings: In this paper, the authors have proposed the perspective that photobiomodulation, through an integrated effect on the neuroimmune and microbiome–gut–brain axis, has the potential to be effective in managing the fatigue, pain, and depressive symptoms of people with inflammatory bowel disease.

## 1. Background

Photobiomodulation (PBM) therapy is the use of defined wavelengths of light/photons in the visible and near-infrared region of the electromagnetic spectrum, at varying irradiances (power output per unit area of treatment) and fluences (photonic energy), to influence molecular and cellular activity and whole-body systems. Typically, PBM therapy is applied using either laser or light-emitting diodes, in contact with or near the skin or mucosal surface, but other novel applications are being explored. Treatment time varies according to the chosen fluence, and the power output of the device being used. Generally, PBM is an athermal event yet there is speculation that it may induce “microscale thermal changes” in cells [1].

Many have described PBM mechanism/s and argued for their identification as a key component to the acceptance by mainstream medicine of PBM. Commonly cited explanations and some hypotheses of how PBM influences biological activity, including condition-specific mechanisms, are listed in Table 1. Despite the myriad propositions regarding mechanism/s, PBM has gained a significant evidence base in the last 10–15 years and is being used and investigated in an ever-increasing number of conditions including those with no gold-standard medical treatment.

The concept of “mechanism” has been described by Nicholson [9] as having three meanings: (1) mechanism defined as “The philosophical thesis that conceives living organisms as machines that can be completely explained in terms of the structure and interactions of their component parts” (p. 153); (2) machine mechanism describing the internal workings of a machine-like entity; and (3) causal mechanism defined as “A step-by-step explanation of the mode of operation of a causal process that gives rise to a phenomenon of interest” (p. 153). We suggest that there may be several co-existing phenomena (including mechanisms) that are activated by the application of PBM to a biological system with sentience, and which cannot necessarily be explained by biophysical causes. For example, if one accepts that health-related quality of life (HRQoL) is the individual’s overall perceived mental and physical health, PBM must have much more than a molecular analgesic mechanism given that HRQoL has been shown to be positively influenced by PBM, e.g., [10].

In this paper, we take a holistic (biopsychosocial or mind–body) approach to PBM. We accept the view that photons activate sub-cellular componentry via photoacceptors. Photoacceptors exist throughout the human body. Made up of interconnecting systems, the human body will respond to photons in numerous, concomitant ways that cannot be broken down, as defined by Nicholson, into discreet component parts or machine-like elements to explain all the effects of PBM. Although causal, process elements will be objectively measurable, there will also be subjective (patient-perceived) outcomes that may not be directly measurable. Such effects include typical domains of HRQoL (i.e., mental, emotional, and social functioning) that may require an understanding of psychological changes, and direct and indirect effects of light on the mind as well as the body.

It is necessary to consider the effects of PBM in an integrated way because most conditions in which PBM has been shown to have efficacy will affect those who suffer from the condition in numerous biopsychosocial ways. For example, a person affected by the persistent pain of severe osteoarthritis of the knee is also likely to experience physical dysfunction. The physical impacts may result in reduced physical activity, resulting in weight gain, subsequent dietary efforts to control weight, and resulting effects on quality of life. If treatment is unsuccessful, knock-on effects of depression, loss of independence, and social isolation can occur. On the other hand, if knee pain can be managed effectively by PBM, the individual may be more willing to exercise, thus contributing to weight loss and the likelihood of improved HRQoL. In this paper, we use the example of inflammatory bowel disease (IBD) expressed as two main subtypes—Crohn’s disease and ulcerative colitis—to demonstrate the need to consider PBM in a holistic way rather than a purely mechanistic way. 

IBD is a chronic, immune-mediated disease of the gastrointestinal tract, frequently expressing extraintestinal involvement, characterised by remissions and relapses [11]. Although not verified definitively, IBD is considered to have four primary causes: abnormal gut microbiota, immune response dysregulation, environmental changes (the “western lifestyle”, which includes physical inactivity, obesity, smoking, and a diet of high saturated fat, processed meats, and a low intake of vegetables, fruit, and fibre), and genetic factors. Recent research into the aetiology and treatment of IBD, formulated IBD as a “complex disease” caused by interacting environmental, genomic, microbial, and immunological factors. The authors introduced the concept of the “IBD interactome”, a biological system comprised of four functionally integrated components (exposome, genome, microbiome, and immunome) and their mutual interactions [12,13]. The conceptualisation of IBD as an interactome explains why IBD cannot be successfully treated by addressing individual disease subunits in isolation but by addressing all its components and their complex interactions [12]. We offer a theoretical perspective for how existing evidence gleaned from other studies may underpin a PBM treatment strategy of potential benefit to people with IBD.

Common presenting features of IBD include abdominal pain, rectal bleeding, diarrhoea, weight loss, anaemia [14], and fatigue [15]. Significant levels of fatigue, depression and abdominal and musculoskeletal pain, which worsen the course of IBD, also impair HRQoL and contribute to a high burden of disease [16,17]. In the next section, we consider the components of the IBD interactome and common signs and symptoms of IBD that may be amenable to PBM.

## 2. PBM and the Microbiome

We propose that the effect of PBM on the microbiome is at least twofold, being a direct effect in abdominal applications, and an indirect effect via the bidirectional microbiome–gut–brain axis. 

Although the specific effect of PBM targeted at inflammation within the gastrointestinal tract has not been investigated, it would seem plausible that it might influence the inflammatory features of IBD. Liebert et al. [18] and Bicknell et al. [19,20] have investigated the effects of PBM on the gut in small clinical studies of people with Parkinson’s disease, confirming pre-clinical animal studies [21] showing that PBM (red and infrared wavelengths) can alter gut microbiome diversity and increase numbers of bacteria associated with a healthy microbiome. There would appear to be sufficient published data to support the notion that PBM may influence the gut microbiome in people with IBD and is thus deserving of further research. If this notion can be established empirically, the result might establish a rationale for PBM treatment of the gut in people with IBD, with the aim of influencing both the microbiome and the concomitant local and systemic inflammation associated with the disease.

## 3. PBM, the Microbiome–Gut–Brain Axis and Hypothalamic–Pituitary–Adrenal Axis

Borovikova et al. [22] described how the vagus nerve exerts anti-inflammatory effects through central activation pathways such as the hypothalamic–pituitary–adrenal (HPA) axis and the peripheral parasympathetic inhibition of proinflammatory cytokines such as TNF, IL-1ß, and IL-6. Laakso et al. [23] proposed that an opioid analgesic effect of peripherally applied PBM may be mediated by centrally derived immune modulators via the HPA axis. In later work, Laakso and co-workers reported mixed effects of PBM on descending inhibitory pathways of the neuroimmune system and of the autonomic nervous system [24,25,26]. Whilst this central analgesic mechanism has been subsumed by later hypotheses regarding PBM mechanisms, the proposal may have some new currency given contemporary investigations regarding the gut–brain axis. Targeting the vagus nerve itself by any external stimulus runs the hypothetical risk of perturbing its myriad other functions, yet, if one considers that the gut–brain axis integrates information from the HPA axis and other parts of the immune system, the stimulation of the vagus at one of its peripheral termini such as the gut may be safe. Vagal nerve dysfunction has been noted in ulcerative colitis [27]. As a gut–brain neurotransmitter, Bankskota and Khan [28] suggest that 5-hydroxytryptamine (5-HT) is the common regulatory signal between many parts of the neuroimmune systems at the local and systemic levels. 5-HT may activate the HPA axis and alter the function of the gastrointestinal tract in people with depression or other psychiatric co-morbidities [29]. Others have reported a relationship, in people with IBD, between the autonomic nervous system, psychological adjustment [30] and inflammation [31]. Should that be the case, PBM applied to the gut may have a role in influencing depression. 

## 4. PBM and the Attenuation of Inflammation (Inflammasome) in IBD

Whereas ulcerative colitis affects only the colon with generalised inflammation, Crohn’s disease can affect any part of the gastrointestinal tract (GIT) and expresses irregularity in presenting areas of inflammation. Several pro-inflammatory cytokines have been implicated in the pathogenesis and progression of IBD, including TNF-α, *IL-6*, and TGF-β (the latter having dual roles in IBD) [32]. 

There is increasing research evidence supporting the efficacy of PBM in attenuating both systemic and local inflammation in a variety of conditions, including immune-mediated diseases such as rheumatoid arthritis, autoimmune thyroiditis, and multiple sclerosis [33]. Specifically, research has shown that PBM can reduce reactive oxidative species (ROS) in oxidatively stressed cells. PBM can up-regulate antioxidant defences, reduce oxidative stress, and decrease inflammatory markers. Arguably, the most commonly cited effect of PBM is a reduction in inflammation, evident in a range of acute and chronic disorders and diseases. Specifically, PBM has been shown to reduce macrophage M1 phenotype markers and reactive nitrogen species and prostaglandins in various animal models, and these effects have been elegantly summarised by Hamblin [33]. 

We therefore propose that the use of PBM directed at the abdomen can down-regulate the local inflammatory response, either through a PBM-mediated increase in microbiome diversity and the resulting activation of the microbiome–gut–brain axis, or a direct effect on mucosal neurons.

## 5. PBM and IBD-Related Pain

The pain of IBD includes inflammatory and non-inflammatory and intestinal and non-intestinal sources, presenting as abdominal pain and episodic peripheral joint pain. Persistent abdominal pain in IBD is generally thought to be nociceptive in nature and commonly results in poor quality of life, depression, and anxiety [34]. There is no treatment for visceral pain. Arthralgia and arthritis of the major joints are commonly associated with a “leaky gut”, for which there appears to be an *HLA* genetic component hypothesised to make patients susceptible to gut microbiota triggering arthritis [35].

A range of medications are used to treat IBD pain, including non-steroidal anti-inflammatory drugs (inclusive of *COX-2* inhibitors), which have been associated with side effects from prolonged use and “flares” in a small percentage of people with IBD [34]. Steroids, anti-TNF-α drugs, and/or immunosuppressants and opiates may assist with the pain of inflammatory arthritis but can also cause significant short- and long-term side effects. Given the significant adverse effects and limited efficacy of these medication groups and addiction issues with opiates, a non-medication treatment alternative would be preferable. 

Although PBM has not yet been investigated for its potential to reduce visceral pain or inflammation in IBD, it has strong evidence in the management of musculoskeletal pain, either alone or in combination with other therapies such as exercise. Briefly, the broad range of wavelengths used in PBM when applied transcutaneously penetrates sufficiently to affect the peripheral nerve endings of nociceptors within the epidermis. Photons, acting as a stimulus, are carried downstream to affect nerves in the subcutaneous tissues, sympathetic ganglia, and the neuromuscular junctions located within muscles and main nerve trunks [36]. Research regarding the analgesic effects of PBM has revealed the activation of multiple pathways and mechanisms including reduced mitochondrial membrane potential, cytoskeletal disruption, and reversible axonal varicosities, resulting in the blockage of fast axonal flow [6,37], the inhibition and/or reduced amplitude of action potentials, and the slowing of nerve conduction velocity [38]. 

Many studies report the efficacy of PBM in achieving significant analgesia and benefits in a variety of acute and chronic orthopaedic and musculoskeletal conditions [39,40], some of which are caused by underlying immune system dysregulation, and some have resulting chronic inflammation. Such conditions include, for example, fibromyalgia [41], knee osteoarthritis [42], neck pain [43], tennis elbow [44], shoulder tendinopathy [45] and other tendinopathies [46], plantar fasciitis [47], and temporomandibular disorder [48]. PBM as a treatment was incorporated into the guidelines for the treatment of neck pain by the World Health Organization’s Committee of the Decade of the Bone and Joint [49], yet there remain disparate findings of effectiveness in other conditions (e.g., lower back pain [50], compared with [51]).

Where pain is caused by tissue damage and the resultant inflammatory response, PBM also has a role [7]. The analgesic efficacy of PBM therapy may be contributed to by its additional effects such as the promotion of wound healing [52,53], skeletal muscle repair [54], and physical performance recovery after exercise [55]. Mechanisms involved include the lowering of levels of reactive oxygen species, the upregulation of antioxidant defences, a reduction in *IL-6* and *IL-10*, the modulation of TNF-α, NF-ĸB, and PGE2, and the modulation of the gene expression of *COX-1* and *COX-2* [33]. The modulation of endogenous opioid production is also proposed in acute and chronic inflammation [23,56,57]. Given that some of these effects are reflective of the inflammatory effects seen in IBD, it would appear reasonable to assume that the use of PBM could influence the inflammatory pain of IBD. 

Knowledge of the specific PBM parameters required for the treatment of pain is emerging with the premise that different combinations of treatment parameters will stimulate different pathways and mechanisms. For example, Holanda and colleagues [37] suggest that neuropathic pain could be treated initially with high irradiance/fluence 808 nm PBM therapy for fast analgesia, and subsequently with low irradiance/fluence for longer-lasting analgesia. The site of application of PBM may not be as critical as once thought. For example, Laakso [58] reported that focused targeting of the vessels providing blood supply to the elbow lateral epicondyle might be necessary for obtaining effective analgesia in lateral epicondylalgia. Yet the notion of an abscopal effect of PBM is gaining traction, whereby the application of PBM in a body area remote to the target tissue can be efficacious but perhaps not as much as when the target tissue is irradiated directly [3].

## 6. PBM, Fatigue, and Depression

The pain and inflammation of IBD, combined with its management by drugs and biologics and the associated systemic changes can result in fatigue. Fatigue, as a symptom rather than a physical sign, can be overlooked in chronic and intractable diseases. Extremely high levels of fatigue have been noted in IBD and there is a dearth of effective, evidence-based treatment [17]. Significant levels and prevalence of fatigue (>80%) are noted in individuals with active IBD, but it improves or disappears with successful treatment [17]. However, 40–46% of patients in remission or with quiescent IBD continue to experience significant levels of fatigue [17,59]. The persistence of fatigue during IBD remission may be related to a number of factors, including continuing low-grade systemic inflammation, IBD medication side effects, nutritional deficiencies, comorbid depression, other comorbidities such as joint pain, respiratory, and cardiovascular disease, and poor sleep quality [17,60].

The fatigue of IBD is associated with depression [60]. Depression can both cause and worsen fatigue through its symptoms (such as the inability to feel pleasure in normally pleasurable activities and reduced energy levels) and through its impact on the course of IBD [16,17,61]. Depression and IBD-related pain are likely to be associated with a number of postulated mechanisms, including underlying inflammatory pathways and the amplification of pain signals brought on by stress [62].

People presenting with fatigue and depression have been shown to have high levels of inflammatory immune activation and other immune system alterations [63]. Generally, fatigue can be categorised into central (neural axis) and peripheral (non-neural axis, e.g., muscular) types. Morris and colleagues [64] elegantly summarised a variety of immune, inflammatory, oxidative and nitrosative stress (O&NS), bioenergetic, and neurophysiological abnormalities involved in the aetiopathology of fatigue, including “…increased levels of pro-inflammatory cytokines, e.g., interleukin-1 (IL-1), *IL-6*, tumour necrosis factor (TNF) α and interferon (IFN) α; O&NS-induced muscle fatigue; activation of the Toll-Like Receptor Cycle through pathogen associated (PAMPs) and damage-associated (DAMPs) molecular patterns, including heat shock proteins; altered glutaminergic and dopaminergic neurotransmission; mitochondrial dysfunctions; and O&NS-induced defects in the sodium-potassium pump. Fatigue is also associated with altered activities in specific brain regions and muscle pathology, such as reductions in maximum voluntary muscle force, downregulation of the mitochondrial biogenesis master gene *peroxisome proliferator activated receptor gamma coactivator 1-alpha*, a shift to glycolysis and build-up of toxic metabolites within myocytes. As such, both mental and physical fatigue, which frequently accompany immune-inflammatory and neuro-inflammatory disorders, are the consequence of interactions between multiple systemic and central pathways” [64] (pp. 1195–1196).

### 6.1. The Potential Role of PBM in Mitigating Peripheral Fatigue

Morris et al. [64] described the effects of ROS in the periphery, including the atrophy of skeletal muscle and the effects on muscle repair, regeneration, and function. PBM has been shown to modulate many factors associated with fatigue. For example, Hamblin [3] noted the beneficial and detrimental modulation of mitochondrial production of ROS by PBM when using different parameters of wavelength and power. Hamblin also noted that secondary mediators of PBM such as ROS, cAMP, and nitric oxide can activate signalling pathways and transcription factors such as inducible NF-kB that play a role in regulating immune responses. In a review paper, the same author noted that pro-inflammatory cytokines TNFα, NF-kB, IL-1, and *IL-6* are reduced using PBM in experimental models [33]. Other studies have demonstrated that PBM attenuates peripheral fatigue, influencing markers of muscle damage, improving exercise performance, and enhancing muscular adaptations to exercise [65]. As pre-conditioning to an exercise protocol, PBM has been shown to have moderate evidence for improving the markers of physical performance (e.g., exercise capacity and time to exhaustion [66] and peak muscle torque [67] and low to moderate evidence for reducing signs of muscle fatigue in healthy subjects [67]). Similar results have been noted more recently in people with chronic obstructive pulmonary disease [68].

Numerous studies of the effects of PBM on physical performance and related measures have shown its beneficial effect on peripheral fatigue indicators. For example, Toma et al. [69] found that PBM significantly reduced muscle fatigue across a range of indicators, including reduced ratings of perceived exertion, increased electromyographic fatigue index, as well increases in muscle peak torque, time to peak torque, total work, average power, and average peak torque, with significant differences between both PBM and placebo conditions. Oliveira et al. [70] found that when compared to placebo, PBM applied before exercise improves indicators of muscle performance, potentially by increasing the local matching of bulk and microvascular oxygen delivery relative to muscle oxygen utilisation. Taken together, these and other results [67,68] suggest that peripheral fatigue in chronic disease could be mitigated using PBM.

### 6.2. Impact of PBM in Alleviating Central Fatigue and Depression

Morris et al. [64] speculate that functional and structural abnormalities in the frontal and prefrontal cortex and the basal ganglia may be the source of central fatigue. These adverse effects are postulated to occur due to pro-inflammatory cytokines and IFNα transported to the brain in the systemic circulation and low oxygen perfusion levels and impaired glucose metabolism. As TNFα, *IL-1β*, and *IL-6* disrupt the blood–brain barrier, they may also induce neuroinflammation and inhibit neurocognitive processes. Morris et al. [64] also note that mitochondrial dysfunction is common in disease states expressing severe or chronic disease where inflammation may be a marker, such as multiple sclerosis, Parkinson’s disease, cancer, and depression some of which have been recently identified as targets for PBM, perhaps due to the effect on mitochondria, e.g., [71,72,73,74]. 

Signals relating to systemic immune-inflammatory responses also reach the nuclei of the solitary tract and the caudal medulla [75] through afferent vagal signals induced by the presence of pro-inflammatory cytokines in lymph nodes and the spleen. Electrostimulation of the vagus nerve has had benefits in treatment-resistant depression [76] with the proposed mechanism via afferent vagal nerve projections to key areas of the brain. Given the enormous interest in the microbiome–gut–brain axis proposed to operate via the vagus nerve, one could propose a connection between gut inflammation from IBD and related fatigue and depression. 

In an alternative hypothesis, recent research [77] has proposed that neurochemical substances released by 5-HT-containing enterochromaffin cells in mouse colon mucosa are likely to disperse onto spinal afferent nerve endings (or their axonal varicosities), then relaying the sensory information to the spinal cord and brain. Chow et al. [6] demonstrated that PBM acts on nociceptor-specific neurons, inducing reversible varicosities indicative of microtubule disruption, the latter likely reducing mitochondrial membrane potential and ATP. Resulting nerve conduction blockade with higher fluences of PBM could potentially operate in concert with 5-HT to orchestrate a beneficial effect of PBM on the gut–brain axis. Such a phenomenon would need to be tested. 

Whilst PBMt has a significant evidence base for modulating physiological markers across a range of conditions, it has recently been shown to have early promise in influencing neuropsychiatric/neuropsychological effects in a range of conditions that may have similar non-intestinal symptomology to IBD. Major depressive disorder (MDD) has been linked to inflammation, including significant increases in chemokines and cytokines including *IL-1α*, *IL-1β*, and *IL-6* [78]. In transcranial applications of PBM, others have demonstrated an increase in ATP production in brain-derived neurons; an improvement in cerebral blood flow and increased regional cerebral blood flow, possibly by the noted increase in nitric oxide and its effect on vasodilation; and a reduction in the expression of cortical TNF-α, *IL-1β*, TGF-β, and *IL-6* [79]. Cassano and colleagues [78] summarised their review of PBM for MDD, stating that PBM “…increases neurotrophins, neurogenesis, synaptogenesis, and ATP, while it reduces inflammation, apoptosis, and oxidative stress”. These and other authors have used PBM in small clinical studies of MDD, finding the reduction and remission of depressive symptoms with transcranial PBM. Cassano et al. [74] identified that PBM was well tolerated by people with depression. In people with Parkinson’s disease, light has been shown to improve sleep [80], this finding being relevant, as people with depression often experience sleep disturbances. A range of symptoms, including sleep, sense of smell, and cognitive disturbance, have been noted to improve in people with Parkinson’s disease exposed to regular and continuing applications of transcranial and abdominal PBM [81].

## 7. PBM, Quality of Life, and Well-Being

HRQoL is severely impacted by IBD [82] and manifests as poor sleep and increased disease severity. Increased levels of physical activity have been associated with improved HRQoL in 158 men and women with IBD [83]. Keller et al. [84] conclude that the social and psychological factors of IBD are underestimated and should be considered important targets for treatment. 

Although HRQoL and overall well-being have not consistently been primary targets of PBM research, based on a random sample of publications, the authors found evidence that HRQoL is responsive to PBM as part of its biopsychosocial effects. For example, in a systematic review and meta-analysis, Camolesi et al. [10] found that there were significant improvements in oral HRQoL in people with burning mouth syndrome. Gautam et al. [85] found that physical, emotional, and functional well-being was significantly better in an active PBM treatment group compared to placebo treatment in people receiving chemoradiotherapy for head and neck cancer. However, social well-being was no different between groups. In a case study of transcranial PBM in a person with Alzheimer’s disease, improvements in HRQoL were reflected in improved measures of caregiver stress and instrumented activities of daily living [86]. In a study of PBM compared to drug therapy, measures of QoL using the Fibromyalgia Impact Questionnaire [87], as well as pain intensity, fatigue, morning stiffness, and depression, were included in a study of people with fibromyalgia, comparing active PBM, placebo PBM, and amitriptyline. Although placebo scores were improved across a number of domains, the authors concluded that amitriptyline and PBM were effective on clinical and HRQoL indicators, but PBM was more effective for pain and fatigue than amitriptyline. Youssef et al. [88] found improvements in the Western Ontario and McMaster Universities Osteoarthritis (WOMAC) index for quality of life in a placebo-controlled dose comparison study in older people with knee osteoarthritis, all of whom undertook a prescribed program of exercise. The placebo PBM group had the least improvement in the WOMAC index.

The above findings are a diverse sample of papers in which HRQoL has been assessed as a patient-reported outcome measure in addition to physical measures. The variety of conditions in which it has been used, and the reported improvements in HRQoL as well as physical indicators, reflect the benefits of PBM on holistic, biopsychosocial factors that are likely to be underpinned by integrated mechanisms of effect, some of which have been explored herein. The effects on the mind may arise from the improvement in an individual’s pathophysiological status, but one should not ignore the potential for PBM to have an effect beyond just physical or molecular mechanisms. Of course, such conjecture needs to be investigated.

## 8. PBM Adverse Reactions 

A drawback of pharmaceutical agents is that they may have adverse side effects and toxicity limiting their efficacy. PBM is remarkably free from adverse effects. In published studies of the abdominal applications of PBM in animals and humans, several authors have noted the absence of adverse effects related to PBM [81,89,90,91]. We do not foresee such factors impacting the use of PBM in people with IBD. However, our research interrogates this phenomenon, as reactions to PBM have previously been described in other applications. Such effects have included short-term/temporary feelings of nausea, faintness, tiredness, weakness, shakiness, euphoria, stomach distension, or an increase in the pain of fibromyalgia [92], and a strange taste in the mouth, headache, decreased appetite, excess sweating, unsteadiness, weight gain, and sleep disturbance in people receiving transcranial PBM in major depressive disorder [93]. None of the effects has been classified as a serious adverse event; indeed, in some cases, we propose such effects may be indicators of potential responsiveness to photonic energy, which, in a clinical setting, may require individualisation of the treatment dose. Moro and colleagues found no evidence of PBM toxicity in their studies of non-human primates [94], and Moskvin and Khadartsev [95] explain why laser PBM has no teratogenic, mutagenic, or carcinogenic effects. 

## 9. Summary and Implications 

**Figure 1:**
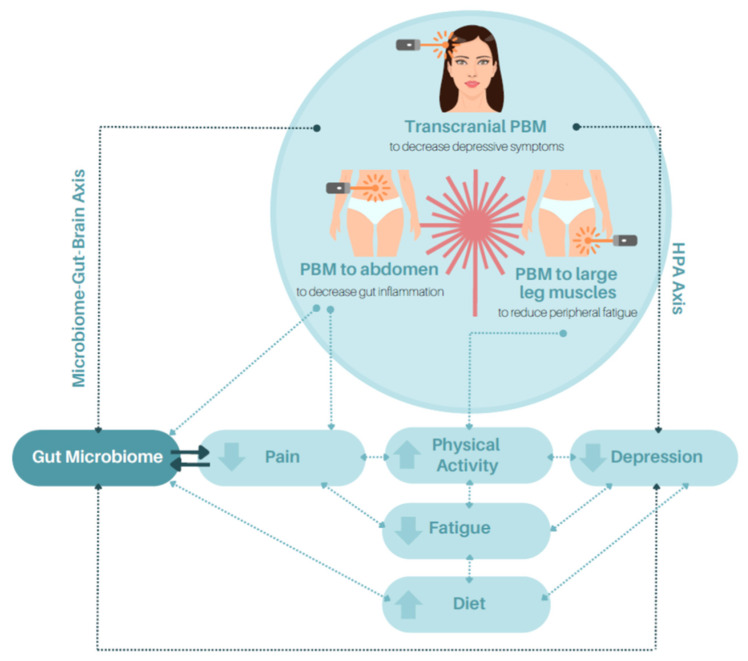
Proposed interactive effects of PBM on fatigue, pain, and depression in people with IBD.

In this paper, we have proposed that PBM may positively affect the key components and pathways within the IBD interactome such as the microbiome, inflammasome, and microbiome–gut–brain axis, as well as common IBD symptoms of fatigue, pain, and depression. We expect that PBM application is needed at multiple sites to obtain benefits across multiple systems affected by IBD (Figure 1). We are investigating this expectation in a current study of young adults with IBD.

We have reviewed the literature that potentially supports our perspective, notably, the contribution of inflammatory cytokines such as TNF-α, *IL-1β*, TGF-β, and *IL-6* to local peripheral, regional, central, and systemic symptomology. Our proposal is founded on the evidence of PBM gleaned from cell culture and animal and clinical models unrelated to IBD. PBM is used for its analgesic effects with an abundance of evidence. For its effects on reducing peripheral (muscular) fatigue, the evidence is building steadily; and for depressive symptoms, early evidence is encouraging. 

This paper demonstrates that there is more than one single mechanism of PBM—it is multifactorial, including machine and causal mechanisms that are likely to have holistic beneficial effects in conditions expressing inflammation. The holistic benefits are likely to include HRQoL and other psychosocial impacts, but these would need to be studied in greater detail. 

We propose that the use of PBM directed at the abdomen to down-regulate the local inflammatory response (either through the activation of the microbiome–gut–brain axis, the HPA axis, or a direct effect on mucosal neurons) and at the large muscles of the legs to up-regulate muscle performance and increase physical activity (by mitigating muscle degeneration from disuse or improving muscle performance characteristics) shall reduce the inflammatory effects of IBD and reduce peripheral fatigue. By reducing inflammation and reducing peripheral fatigue, we propose that the related centrally mediated effects of inflammation, these being depression and central fatigue, will be ameliorated via the direct effect of PBM at the local level or through the neuroimmune system. Our first step (subject of a protocol paper under review) is to test 904 nm laser PBM using parameters that one of the authors (LL) has used previously with demonstrated effects on the microbiome [20] and on muscle performance characteristics [69,70]. Several measures of outcome including the microbiome profile, faecal studies, and participant-reported outcome measures will be used. Depending on the results of our initial pilot work, in which participants are acting as their own controls, we expect to proceed to a randomised and sham-controlled clinical trial. Later work may extend to the inclusion of an integrated home treatment protocol. We may also include the transcranial application of PBM to potentiate the effects of remote PBM treatment on the features of central fatigue and depression. Should these effects be demonstrated in future research, a flow-on effect in participants may be greater appetite and improved diet, which, in turn, would have impacts on overall well-being (Figure 1).

As part of plans for future research, we acknowledge that factors associated with PBM “dosing” will need to be determined. For example, in our world-first current research, we have noted the varying body size of participants with IBD and suggest that there may be a need to individualise the number of PBM application points at the abdomen and legs. The regularity of treatment will require future research. In the current study, we have chosen to treat our participants once/week in clinic visits. Should our initial pilot work demonstrate the proof of concept, it is likely that more regular home treatments will be necessary. Treatment frequency can be tested in a similar way to author LL’s collaborative work on Parkinson’s disease [96], in which different treatment regimens were tested initially for 4 weeks, and the regimens with the greatest promise were translated later into longer periods of clinical treatment, e.g., [81,91]. People of smaller stature and body morphology (ectomorphs) may require lower fluence and irradiance than endomorphs, in a similar way to how some pharmaceuticals are prescribed according to body weight. Different wavelengths may be required at different body locations to optimise the effects we expect to see. Moreover, if benefits are demonstrated, more research will be needed to understand the specific mechanisms involved. Finally, if PBM is demonstrated to have benefits for IBD, we expect that it would be as an adjunct, not as a replacement for accepted medical treatments.

Clinically, the authors have seen early evidence of reduced fatigue in young people with IBD after treatment with PBM of the abdomen and large lower limb muscle groups. As the effects of inflammation are complex and interrelated, we expect that a multidimensional approach to PBM application will also have beneficial effects on other biopsychosocial facets of the whole being, including quality of life and physical activity. In general terms, we approach the notion that PBM may result in reduced pain and inflammation associated with IBD, which may result in decreased feelings of fatigue. With less fatigue and pain, people with IBD should be more physically active, thus further reducing fatigue and improving functional performance. By increasing physical activity, pain and fatigue are expected to improve, and depression should also be positively influenced. The concomitant inclusion of transcranial PBM may also positively influence depression associated with IBD.

## Figures and Tables

**Table 1:** Proposed mechanisms of PBM.

Mitochondrial chromophores such as cytochrome C oxidase (CCO) accept photonic energy resulting in dissociation of nitric oxide, production of reactive oxygen species, increased mitochondrial membrane potential, increased intracellular ATP and cyclic AMP, changes in Ca^2+^ concentration and numerous downstream effects such as transcription factor activation (NF-κB, HIF-1α and RANKL), e.g., [1,2,3].
Hypothesised modulation of ion channels and protein conformational transfer [4] requires further investigation.
Interaction of far and near-infrared photons with bound mitochondrial water [2,5] refuting the CCO mechanism noted above.
Disruption of the neural cytoskeleton to explain the modulatory effect of PBM in pain [6] with the finding that 830 nm (continuous wave) laser PBM decreased mitochondrial membrane potential, induced reversible varicosity formation in rat dorsal root ganglion neurons blocking fast axonal flow thus explaining clinically observed analgesia via neural blockade.
Modulation of genes, neurotrophins, cytokines, and inflammatory processes in conditions expressing inflammation [2,7]. Examples include TNF, BDNF, several interleukins, VEGF, histamine, and prostaglandins.
Potential *TRPV1* interaction with the cytoskeleton in conditions expressing pain, inflammation, and skin wounds [1,2,8].
Activation of extra-cellular transforming growth factor beta (TGFβ) in tissue healing settings [2].

## Data Availability

Not applicable.
